# The Impact of Embryo Storage Time on Pregnancy and Perinatal Outcomes and the Time Limit of Vitrification: A Retrospective Cohort Study

**DOI:** 10.3389/fendo.2021.724853

**Published:** 2021-10-25

**Authors:** Mengge Cui, Xiyuan Dong, Shuhao Lyu, Yu Zheng, Jihui Ai

**Affiliations:** ^1^ Reproductive Medicine Center, Tongji Hospital, Tongji Medical College, Huazhong University of Science and Technology, Wuhan, China; ^2^ College of Medicine, The Ohio State University, Columbus, OH, United States

**Keywords:** frozen-thawed embryo transfer, embryo vitrification, storage time, pregnancy and perinatal outcomes, time limit

## Abstract

**Background:**

The technique of embryo cryopreservation has been increasingly applied in clinical settings. However, there has been a concern about the safety and efficacy of long-term freezing of embryos. Therefore, the aim of this study was to evaluate whether storage time of vitrification had any effects on pregnancy as well as perinatal outcomes, further, to explore the appropriate time limit of vitrification.

**Methods:**

The study included women who underwent at least one frozen-thawed cycle with single embryo transfer between January 1^st^, 2016 and September 30^th^, 2019. Patients were assigned into 3 groups according to the storage time (<3 months, 3-12 months and >12 months) to evaluate the impact of embryo storage time on pregnancy and perinatal outcomes. To further investigate the time limit of vitrification, propensity score matching was used to compare the primary outcomes of patients with storage time of 1-3 years, 3-5 years, and >5 years to those stored for ≤1 year.

**Results:**

A total of 9806 frozen-thawed embryo transfer cycles were included in our study. After adjustment for confounding variables, no significant differences were found in pregnancy outcomes among groups. However, postponement of transfer increased the risks of large for gestational age and placenta previa. In addition, after propensity score matching, 171 cycles with storage time >5 years were matched with those ≤1 year, both the clinical pregnancy rate and live birth rate decreased significantly when the storage time exceeded 5 years.

**Conclusions:**

The duration of vitrification did not significantly affect the pregnancy outcomes within 5 years period. However, the clinical pregnancy rate and live birth rate both decreased significantly when the duration of vitrification exceeded 5 years. It is worth noting that the conclusion was drawn from a small sample study after propensity score matching and should be treated with caution. In addition, the cycles were from different time periods, which could have an impact on the results.

## Introduction

Embryo cryopreservation, an important derivative of Assisted Reproductive Technology (ART), refers to the technique of keeping embryos in an ultra-low temperature environment (liquid nitrogen, -196°C) for preservation through a series of protective measures, freezing procedures and thawing for transfer when needed (1). The application of such technique has widened significantly since the first live birth after a frozen-thawed embryo transfer (FET) in 1983 (2).

Embryo cryopreservation technique increases the embryo utilization rate, which subsequently improves the cumulative pregnancy rate in an ovarian stimulation cycle. In addition, FET has the advantages of decreasing the risk of ovarian hyperstimulation syndrome (OHSS) (3, 4) and avoiding the negative effect to endometrium receptivity (5, 6), embryo implantation and placentation (7–9) of controlled ovarian hyperstimulation (COH). In recent years, embryo cryopreservation mainly includes two techniques, programmed freezing and vitrification. Compared with the former, vitrification yields higher survival rate and better clinical outcomes (10). During the process of vitrification, embryos are fully dehydrated without formation of crystal, which significantly reduces potential damages to the embryos (11). In addition, with characteristics of simplicity, convenience and cost-efficiency, vitrification has made its way into the mainstream clinical practices. Although the advantages of vitrification are clear, there has also been an emerging concern of embryo safety due to exposure to high-concentration cryoprotectants and long-term contact with liquid nitrogen (12). High-concentration cryoprotectants may cause biochemical and osmotic toxicity and the “open system” makes the embryos contact with liquid nitrogen directly during storage. Therefore, we hypothesize that long-term vitrification has a negative effect on embryos.

Some retrospective studies showed that the length of storage time did not impact the survival rate, implantation rate, pregnancy rate, live birth rate nor birth defect rate (13–16). However, another study insisted that prolonged storage time of vitrification negatively affected pregnancy outcomes (17). In addition, experts in China have discussed and suggested that cryopreserved embryos should be preferably used within 5 years (1). The lack of clinical evidence and the absence of exact guidelines make the issue about appropriate time limit of cryopreservation extremely controversial. Therefore, the aim of this study was to illustrate whether storage time of vitrified embryos had any effects on either pregnancy or perinatal outcomes, and to explore the appropriate time limit of vitrification.

## Materials and Methods

### Study Population and Design

A retrospective cohort study, including all women who underwent at least one frozen-thawed cycle with single embryo transfer, was conducted at the Reproductive Medicine Center of Tongji hospital between January 1^st^, 2016 and September 30^th^, 2019. Exclusion criteria were: non-vitrification; preimplantation genetic testing (PGT); uterine malformation; frozen or donated oocyte, frozen or donor sperm; repeated-frozen-thawed embryo; loss to follow-up, artificial abortion for personal reasons. Finally, 9806 cycles were included for final analysis.

**Figure 1:**
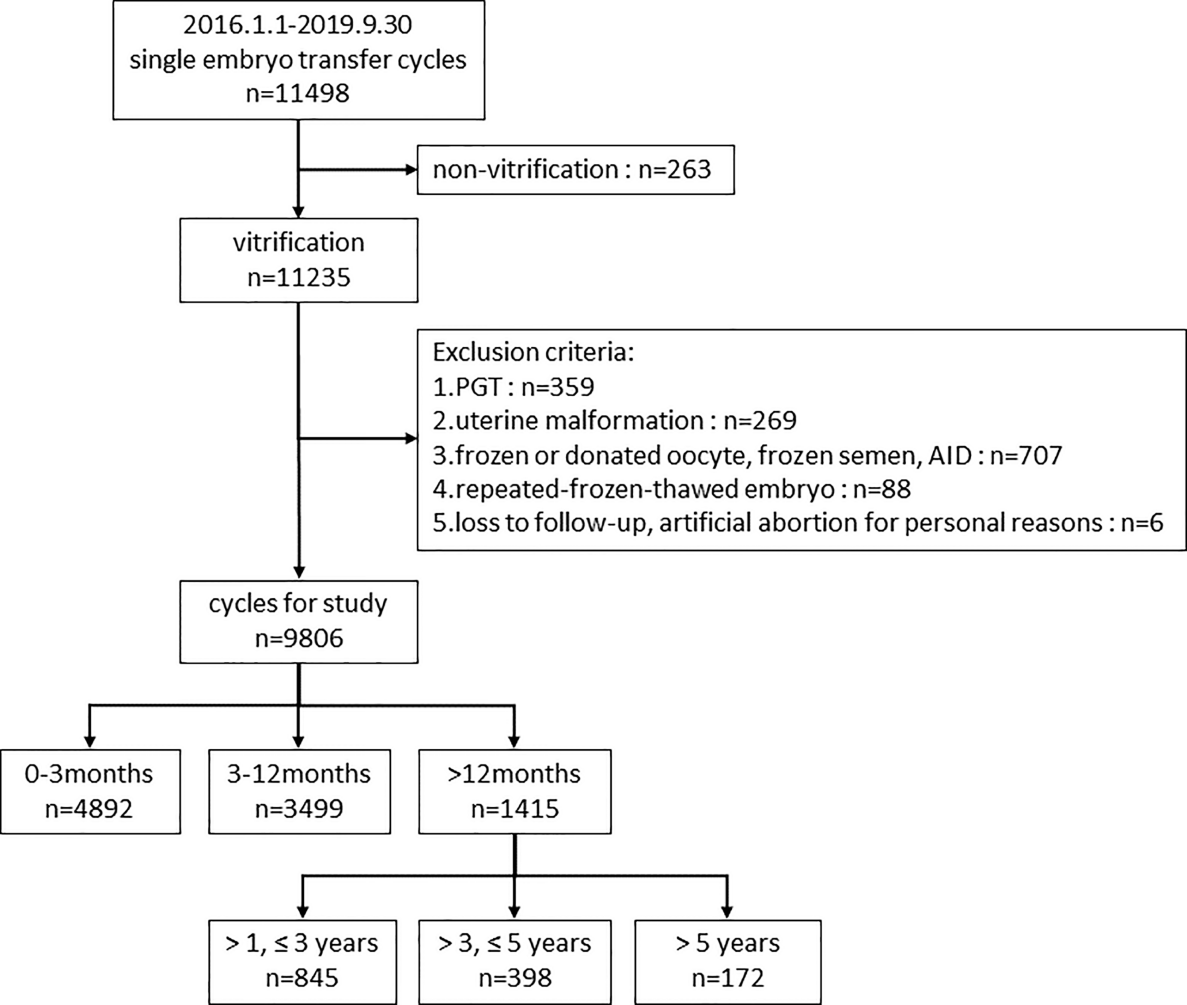
Flow chart of the study design. PGT, preimplantation genetic testing; AID, artificial insemination with donor sperm.

First, we divided 9806 cycles into three groups according to the storage time (<3 months, 3-12 months and >12 months) to study the impact of embryo storage time on pregnancy and perinatal outcomes. Second, to further investigate the effect of prolonged vitrification (>12 months) on pregnancy outcomes and to explore the appropriate time limit of vitrification, we compared the patients with storage time of 1-3, 3-5, and >5 years to those with storage time ≤1 year. The flow chart of the study design was displayed in **Figure 1**.

This study involving human participants was reviewed and approved by the Institution Review Board (IRB) of Tongji Hospital (TJ-IRB20210308). The patients/participants provided their written informed consent to participate in this study.

### Ovarian Stimulation Protocol

Ovarian stimulation protocols were performed in accordance with previously described (18). Briefly, ovarian stimulation was conducted individually by using exogenous follicle-stimulating hormone (FSH) (Gonal-F, Merck Serono, Switzerland) in combination with gonadotrophin-releasing hormone (GnRH) agonists (Diphereline, Ipsen or Decapeptyl) (Ferring Pharmaceuticals, Saint-Prex, Switzerland) or antagonist (Cetrotide, Merck, USA). Recombinant human chorionic gonadotrophin (hCG) (250 mg, Ovidrel, Merck Serono, Darmstadt, Germany) was administered subcutaneously when two or three leading follicles reached a mean diameter ≥18 mm. Oocytes were retrieved 36 hours after hCG triggering. * In vitro* fertilization (IVF) or intracytoplasmic sperm injection (ICSI) was performed as appropriate with respect to semen quality.

### Embryo Culture, Vitrification and Thawing

The procedures of sperm preparation, IVF and embryo culture were described previously (19). Briefly, oocytes were incubated in G-IVF medium (Vitrolife, Sweden) after retrieval and were fertilized 4 hours later. Normal fertilization, assessed at 16-18 hours after insemination, was defined as zygotes with two pronuclei (2PN). Then, fertilized oocytes were continuously cultured in G1 medium (Vitrolife, Sweden) for 2 more days. Embryos were continuously cultured in G2 medium (Vitrolife, Sweden) to Day 5/6.

Morphological evaluation of the embryos on Day 2/3 was implemented according to the Istanbul consensus (20). Available embryos, which were scored as top, good and fair-quality, were transferred or cryopreserved. Blastocyst morphology classification was evaluated using the Gardner scoring system (21) based on the stage of blastocyst (stage 1-6), inner cell mass (score A/B/C) and trophectoderm cells (score A/B/C). Blastocysts better than grade 3CC were used for transfer or vitrification.

Cryotop device and commercial vitrification solutions (Kitazato, Japan) were used for embryos vitrification. The procedure was performed as previously described (19). Briefly, embryos were firstly equilibrated in equilibration solution for 5–10 minutes. Subsequently, embryos were put into vitrification medium and immediately loaded onto the surface of the cryotop with a minimum volume. The whole procedure was completed within 1 minute before the sample was submerged in liquid nitrogen. On the day of embryo transfer, embryos were first transferred to 37°C thawing solution for 1 minute, followed by 3 minutes in diluent solution and then washed twice in washing solution for 5 minutes respectively at room temperature. Warmed embryos were then cultured for at least 2 hours prior to further evaluation.

### Endometrial Preparation and Embryo Transfer

According to the situation of patients’ menstrual cycles, appropriate endometrial preparation method was selected. Usually, a natural cycle was used for patients who had regular menstrual cycles, an artificial cycle for those with irregular menstrual cycles. Only one embryo was transferred. Whether to transfer a cleavage embryo or a blastocyst depended on women’s age as well as the quality and the number of available embryos individually. All patients underwent routine luteal phase support after transfer.

### Outcome Parameters

The primary outcome measures of our study were clinical pregnancy rate (CPR) and live birth rate (LBR). Secondary outcome measures included biochemical pregnancy rate, multiple pregnancy rate, ectopic pregnancy rate, miscarriage rate and perinatal outcomes.

A clinical pregnancy was confirmed by the observation of a gestational sac *via* ultrasound examination approximately 5 weeks after embryo transfer. A live birth was defined as delivery of a living baby after 28 weeks of gestation. A biochemical pregnancy was defined as a pregnancy diagnosed only by the increase of serum hCG, which was detected 14 days after embryo transfer, but without a gestational sac. A multiple pregnancy was defined as more than one intrauterine fetus simultaneously. Ectopic pregnancy was diagnosed by ultrasound or by laparoscopic visualization of at least one extrauterine gestational sac. Miscarriage was defined as the loss of fetal heart activity within 20 weeks after confirming clinical pregnancy.

Only the women with singleton live birth were included in the calculation of perinatal outcomes. The perinatal outcomes included gestational age, preterm birth (<37 weeks’ gestation), early preterm birth (<32 weeks’ gestation), birth weight, small for gestational age (SGA), appropriate for gestational age (AGA), large for gestational age (LGA), delivery mode, newborn gender, gestational hypertension, gestational diabetes mellitus, placenta previa and birth defects. The determination of SGA and LGA was based on the birthweight reference for Chinese populations (22). SGA was defined as birth weight lower than the 10th percentile of referential birthweight. LGA was defined as birth weight higher than the 90th percentile of referential birthweight. Birth weight between 10th and 90th percentile was regarded as AGA.

### Statistical Analysis

Descriptive statistical analysis was performed on basic demographic characteristics, pregnancy outcomes and perinatal outcomes. Continuous variables were presented as the mean ± standard deviation (SD) and analyzed by ANOVA test for normally distributed ones, or demonstrated as medians and interquartile ranges (IQRs) and tested with Kruskal-Wallis test when variables showed a non-normal distribution. Categorical variables were presented as counts and percentages, and were compared using the Chi-square test or Fisher’s exact test when appropriate.

To investigate the effect of storage time on pregnancy and perinatal outcomes, multivariable logistic regression analyses were conducted. In addition, multivariable linear regression was used to explore the effect of storage time on gestational age and birth weight. The variables included maternal age at ovum pick-up (OPU), maternal body mass index (BMI), type of infertility, length of infertility, main causes, number of oocytes retrieved, development stage of embryo transferred, number of previous thawing, endometrial preparation method and thickness of endometrium. Univariate analysis was performed to evaluate the effect of each variable on pregnancy and perinatal outcomes. The variables with p value <0.10 were included in the multivariate regression model.

Due to the obvious gap in sample size between the groups ≤1 year (8391 cycles) and 1-3 years (845 cycles), 3-5 years (398 cycles), >5 years (172 cycles), we used propensity score matching respectively to identify the most comparable cycles. The baseline characteristics, including maternal age at OPU, maternal BMI, type of infertility, length of infertility, main causes, number of oocytes retrieved, development stage of embryo transferred, number of previous thawing, endometrial preparation method and thickness of endometrium, were 1:1 matched using the nearest neighbor matching, with a caliper width of 0.1 and without replacement. After propensity score matching, the effect of storage time on pregnancy outcome was further assessed by logistic regression model.

Two-sided p values <0.05 were considered statistically significant. All analyses were performed using SPSS (Version 25.0, IBM, USA) and R software (Version 4.0.3).

## Results

### The Impact of Embryo Storage Time on Pregnancy and Perinatal Outcomes

In this retrospective study, we ultimately included 9806 cycles for analysis. The patients were grouped according to storage time.

Storage time <3 months, 4892 cycles; storage time 3-12 months, 3499 cycles; storage time >12 months, 1415 cycles.

#### Baseline Characteristics

Baseline characteristics of the patients are presented in **Table 1**. The BMI and infertility causes were similar among the three groups.

**Table 1:** Baseline characteristics of single vitrified embryo transfer cycles.

	0-3 m	3-12 m	>12 m	p value		
				p	p1	p2
**number of cycles, n**	4892	3499	1415			
**maternal age at OPU, median (IQR)**	31 (28~36)	32 (28~36)	30 (27~33)	<0.001	0.330	<0.001
**maternal BMI, median (IQR)**	21.5 (19.7~23.5)	21.5 (19.8~23.6)	21.6 (19.8~23.7)	0.237	0.360	0.300
**type of infertility, n (%)**				0.004	0.003	0.261
primary	2835 (58.0)	1900 (54.3)	794 (56.1)			
secondary	2057 (42.0)	1599 (45.7)	621 (43.9)			
**length of infertility, median (IQR)**	3 (2~4)	3 (2~5)	3 (2~5)	0.002	0.611	0.002
**main infertility causes, n (%)**				0.091	0.236	0.139
female factor						
tubal	2538 (51.9)	1841 (52.6)	833 (58.9)			
ovulation	1082 (22.1)	769 (22.0)	372 (26.3)			
DOR	1096 (22.4)	658 (18.8)	157 (11.1)			
EMT	465 (9.5)	364 (10.4)	126 (8.9)			
others	958 (19.6)	743 (21.2)	199 (14.1)			
male factor	466 (9.5)	356 (10.2)	149 (10.5)			
male and female factors	547 (11.2)	347 (9.9)	142 (10.0)			
unexplained	184 (3.8)	117 (3.3)	35 (2.5)			
**number of oocytes retrieved, median (IQR)**	12.0 (6.3~18.0)	11.0 (7.0~16.0)	14.0 (10.0~19.0)	<0.001	0.040	<0.001
**development stage of embryo transferred, n (%)**				<0.001	0.065	<0.001
cleavage embryo	1031 (21.1)	679 (19.4)	183 (12.9)			
blastocyst	3861 (78.9)	2820 (80.6)	1232 (87.1)			
**number of previous thawing, n (%)**				<0.001	<0.001	<0.001
0	4655 (95.2)	2396 (68.5)	949 (67.1)			
≥1	237 (4.8)	1103 (31.5)	466 (32.9)			
**endometrial preparation method, n (%)**				<0.001	<0.001	<0.001
artificial	4281 (87.5)	2746 (78.5)	1166 (82.4)			
natural	214 (4.4)	231 (6.6)	100 (7.1)			
others	397 (8.1)	522 (14.9)	149 (10.5)			
**thickness of endometrium, median (IQR)**	9.2 (8.5~10.0)	9.0 (8.3~10.0)	9.0 (8.3~10.0)	<0.001	<0.001	<0.001

OPU, ovum pick-up; BMI, body mass index; DOR, diminished ovarian reserve; EMT, endometriosis; IQR, interquartile range.

p: group (0-3 m) vs. group (3-12 m) vs. group (>12 m), p1: group (0-3 m) vs. group (3-12 m), p2: group (0-3 m) vs. group (>12 m).

p1 and p2 were the corresponding p values adjusted by the method “False Discovery Rate (fdr)”.

The maternal age, number of oocytes retrieved, length of infertility and endometrial thickness were significantly lower in the patients with storage time >12 months, compared with the patients with storage time <3 months and 3-12 months. The proportion of primary infertility in patients with storage time <3 months was higher than patients with storage time 3-12 months. The percentage of blastocyst transfer was higher in patients with storage time >12 months, compared with patients with storage time <3 months and 3-12 months. Besides, the distribution of endometrial preparation method varied among the three groups.

#### Pregnancy Outcomes

**Table 2** shows the pregnancy outcomes of the three groups. Referring our primary outcome measures, there were no significant differences among groups in CPR (47.7% vs. 47.1% vs. 50.5%, p=0.089) and LBR (37.7% vs. 36.4% vs. 38.5%, p=0.287). In addition, no significant differences were found in survival rate, biochemical pregnancy rate, multiple pregnancy rate, ectopic pregnancy rate and miscarriage rate.

**Table 2:** The results of pregnancy outcomes.

	0-3 m	3-12 m	>12 m	p value		
				p	p1	p2
**survival rate, %**	99.5	99.4	99.3	0.464		
**clinical pregnancy rate, n (%)**	2333 (47.7)	1649 (47.1)	715 (50.5)	0.089	0.627	0.096
**live birth rate, n (%)**	1842 (37.7)	1272 (36.4)	545 (38.5)	0.287	0.350	0.577
**biochemical pregnancy rate, n (%)**	310 (6.3)	211 (6.0)	85 (6.0)	0.812	1.000	1.000
**multiple pregnancy rate, n (%)**	58 (1.2)	48 (1.4)	20 (1.4)	0.680	0.878	0.878
**ectopic pregnancy rate, n (%)**	25 (0.5)	14 (0.4)	7 (0.5)	0.756	1.000	1.000
**miscarriage rate, n (%)**	123 (2.5)	76 (2.2)	46 (3.3)	0.090	0.346	0.234

OR, odds ratio; CI, confidence interval.

p: group (0-3 m) vs. group (3-12 m) vs. group (>12 m), p1: group (0-3 m) vs. group (3-12 m), p2: group (0-3 m) vs. group (>12 m).

p1 and p2 were the corresponding p values adjusted by the method “False Discovery Rate (fdr)”.

**Figure 2:**
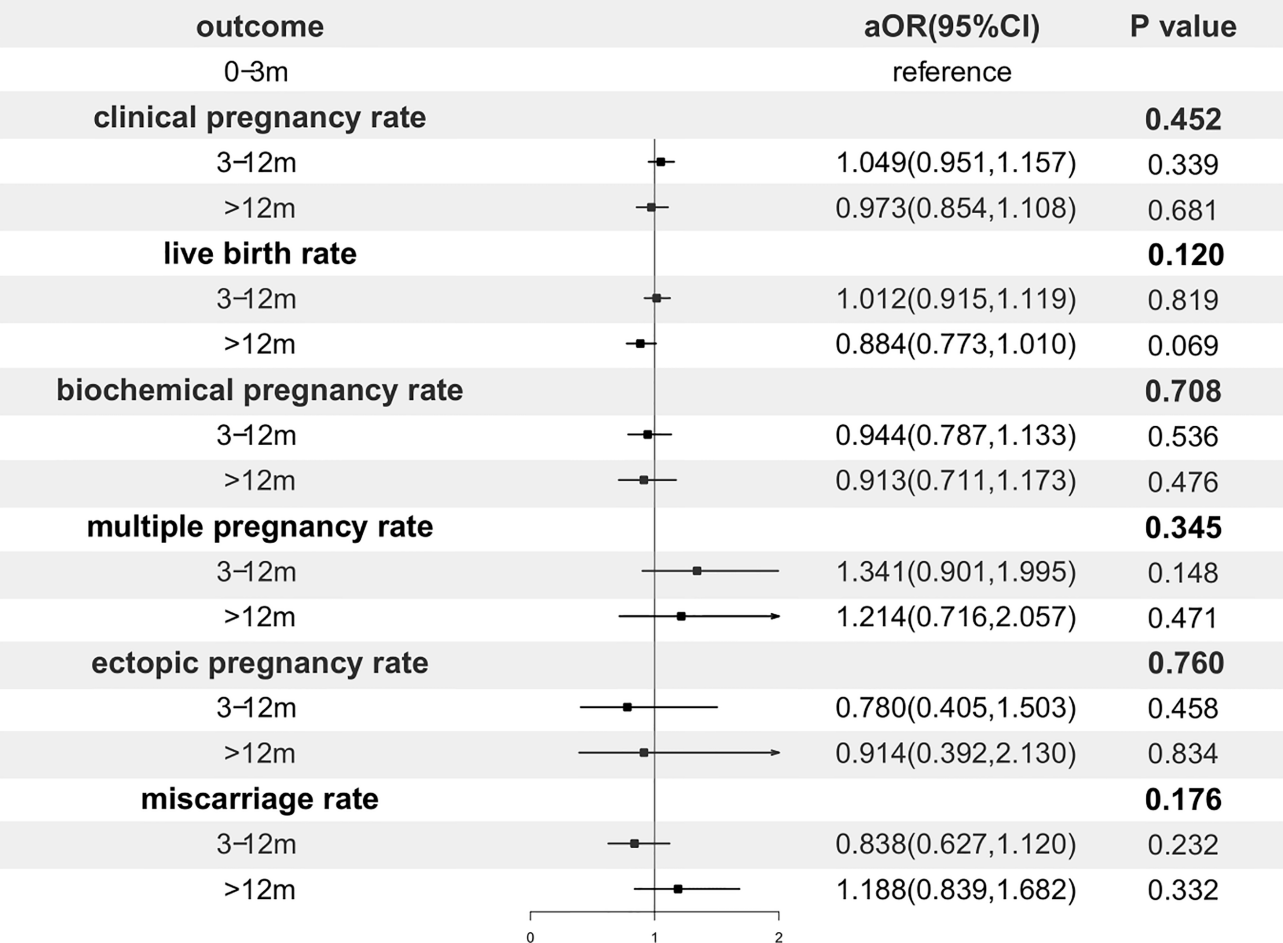
Forest plot of adjusted odds ratio of pregnancy outcomes. OR, odds ratio; CI, confidence interval.

In order to adjust confounding factors, a multivariable regression analysis was performed and presented in **Figure 2**. After adjustment, the storage time had no significant effect on CPR (p=0.452), LBR (p=0.120) and any of the other pregnancy outcomes.

#### Subgroup Analysis of Primary Outcomes According to Maternal Age at OPU

The subgroup analysis was performed split by maternal age at OPU (<30, 30-35, ≥35 years old) in **Supplementary Stable 1**. In the subgroup of maternal age at OPU <30 years old, the storage time had no significant effect on CPR (p=0.132) and LBR (p=0.336) after adjustment. In the subgroup of maternal age at OPU 30-35 years old, the storage time was not associated with CPR (p=0.849) and LBR (p=0.505) after adjustment. In the subgroup of maternal age at OPU ≥35 years old, no correlations were found between the storage time and CPR (p=0.102) or LBR (p=0.164) after adjustment.

#### Perinatal Outcomes

Of all 9806 cycles, 3602 cycles with a singleton live birth were used to analyze perinatal outcomes. **Table 3** shows the details. The gestational age decreased with the storage time, and pairwise comparisons showed that these differences were all significant (mean: 271.5 vs. 270.5 vs. 268.8, p<0.001). The percentage of preterm birth and early preterm birth, birth weight and newborn sex ratio were comparable among all three groups. Cross compared patients with storage time <3 months and 3-12 months (25.2%, p=0.008 and 24.4%, p=0.008, respectively), cryopreservation > 12 months (31.4%) was determined to associate with higher risk of LGA. In addition, the proportion of cesarean section in patients with storage time 3-12 months (90.2%) was significantly higher than those of <3 months (87.2%, p=0.040) and >12 months (86.6%, p=0.049). Regarding gestational diabetes mellitus and birth defects, no significant differences were observed across groups. However, a marginal significant difference was noted in gestational hypertension, and we found a lower incidence in patients with storage time > 12 months (2.2%) compared with those with <3 months and 3-12 months (4.2% and 4.7%). Added, the incidence of placenta previa in group 3-12 months (6.0%) was statistically higher than those in group of <3 months (4.0%, p=0.049).

**Table 3:** Perinatal outcomes of singletons born after single vitrified embryo transfer cycles.

	0-3 m	3-12 m	>12 m	p value		
				p	p1	p2
**number of cycles, n**	1812	1252	538			
**gestational age (d), median (IQR)**	273 (268~278)	273 (266~277)	271 (266~274)	<0.001	<0.001	<0.001
preterm birth, n (%)	119 (6.6)	93 (7.4)	43 (8.0)	0.441	0.592	0.592
early preterm birth, n (%)	14 (0.8)	12 (1.0)	6 (1.1)	0.719	0.963	0.963
**birth weight (g), median (IQR)**	3400 (3100~3700)	3360 (3100~3650)	3400 (3100~3700)	0.097	0.240	0.260
SGA, n (%)	84 (4.6)	57 (4.6)	19 (3.5)	0.536	0.984	0.590
AGA, n (%)	1271 (70.1)	889 (71.0)	350 (65.1)	0.036	0.635	0.043
LGA, n (%)	457 (25.2)	306 (24.4)	169 (31.4)	0.006	0.654	0.008
**delivery mode, n (%)**				0.021	0.040	0.781
vaginal delivery	232 (12.8)	123 (9.8)	72 (13.4)			
cesarean section	1580 (87.2)	1129 (90.2)	466 (86.6)			
**newborn gender, n (%)**				0.649	0.711	0.711
male	1017 (56.1)	712 (56.9)	314 (58.4)			
female	795 (43.9)	540 (43.1)	224 (41.6)			
**complication, n (%)**						
gestational hypertension	76 (4.2)	59 (4.7)	12 (2.2)	0.049	0.550	0.072
gestational diabetes mellitus	110 (6.1)	98 (7.8)	30 (5.6)	0.091	0.166	0.748
placenta previa	73 (4.0)	75 (6.0)	29 (5.4)	0.041	0.049	0.322
birth defects	40 (2.2)	32 (2.6)	15 (2.8)	0.686	0.904	0.904

SGA, small for gestational age; AGA, appropriate for gestational age; LGA, large for gestational age; IQR, interquartile range.

p: group (0-3 m) vs. group (3-12 m) vs. group (>12 m), p1: group (0-3 m) vs. group (3-12 m), p2: group (0-3 m) vs. group (>12 m).

p1 and p2 were the corresponding p values adjusted by the method “False Discovery Rate (fdr)”.

**Figure 3:**
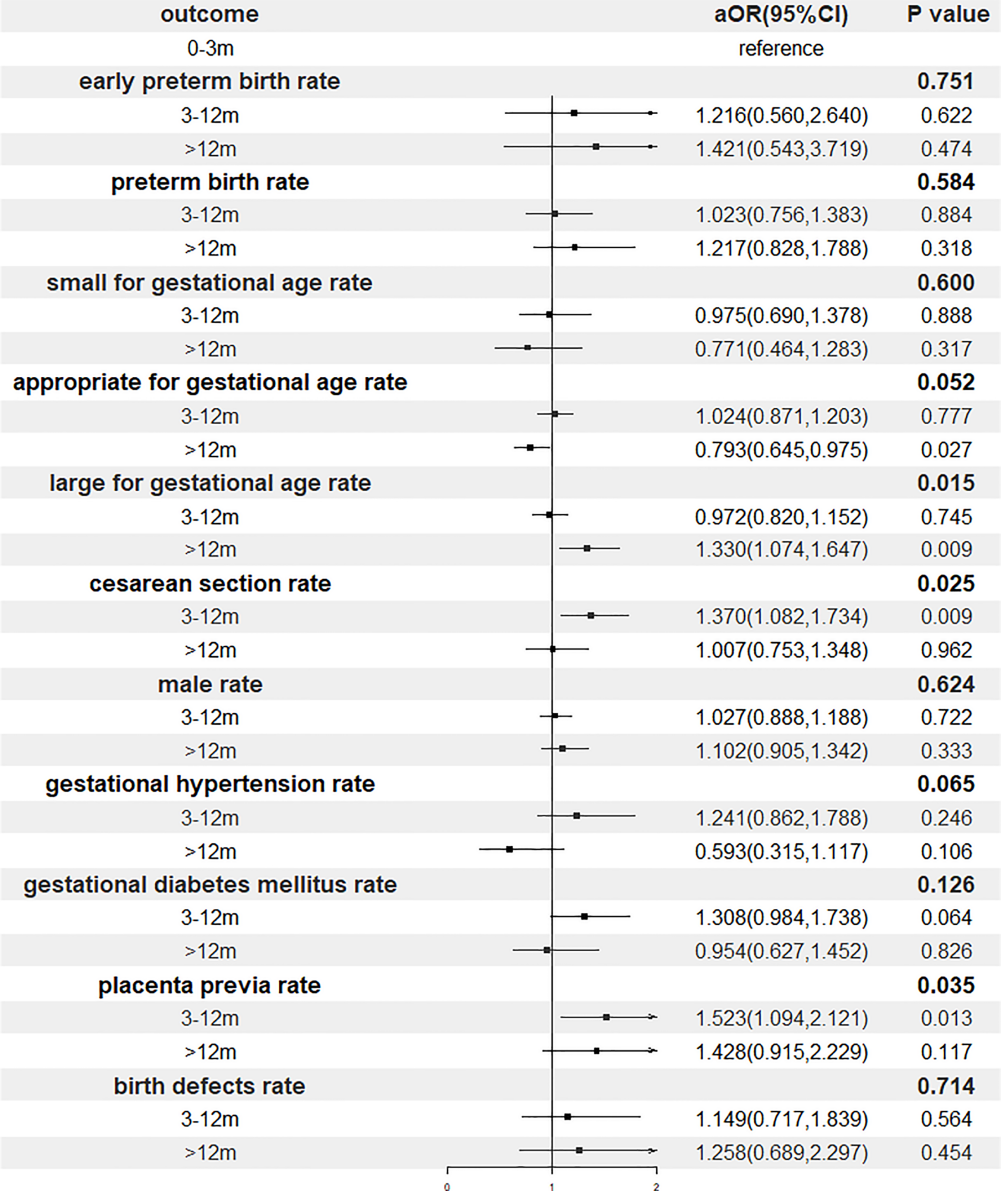
Forest plot of adjusted odds ratio of perinatal outcomes. OR, odds ratio; CI, confidence interval.

Results of the multivariable regression analyses investigating the association of storage time and perinatal outcomes were presented in **Figure 3** and **Table 4**. After adjustment, gestational age, the proportion of cesarean section, the risks of LGA and placenta previa remained increased significantly with postponement of transfer, whereas the relationship between the storage time and gestational hypertension did not reach a statistical significance.

**Table 4:** Multivariable linear regression of gestational age and birth weight.

	0-3 m	3-12 m			>12 m		
		B	Standard error	p value	B	Standard error	p value
**gestational age**							
crude	ref	-1.013	0.413	0.014	-2.577	0.553	<0.001
adjusted	ref	-0.700	0.436	0.108	-2.576	0.575	<0.001
**birth weight**							
crude	ref	-26.751	18.688	0.152	22.864	25.024	0.361
adjusted	ref	-6.364	19.740	0.747	27.269	26.026	0.295

### The Time Limit of Vitrification

#### Baseline Characteristics Before and After Matching

The baseline characteristics of the Group ≤1 year and Group 1-3, Group 3-5 years before and after propensity score matching are summarized respectively in **Supplementary STable2** and **STable3**. **Table 5** presents the baseline characteristics before and after matching between the Group ≤1 year and Group >5 years. Before matching, the Group >5 years showed lower maternal age at OPU (28 (IQR 26~30) vs. 32 (IQR 28~36), p<0.001), greater number of oocytes retrieved (17 (IQR 12~20) vs. 12 (IQR 7~17), p<0.001), longer infertility duration (3.0 (IQR 2.0~5.0) vs. 3.0 (IQR 2.0~4.5), p=0.014) and more blastocysts transfers (90.1% vs. 79.6%, p=0.001). After matching, 171 cycles remained in each group with no significant difference.

**Table 5:** Baseline characteristic of cycles with storage time ≤1 year and >5 years before and after matching.

storage time	unmatched			matched		
	≤1 y	>5 y	p value	≤1 y	>5 y	p value
**number of cycles, n**	8391	172		171	171	
**maternal age at OPU, median (IQR)**	32 (28~36)	28 (26~30)	<0.001	28 (26~30)	28 (26~30)	0.674
**maternal BMI, median (IQR)**	21.5 (19.8~23.6)	21.0 (19.8~23.2)	0.207	21.0 (19.5~23.4)	21.0 (19.8~23.2)	0.829
**type of infertility, n (%)**			0.441			0.745
primary	4735 (56.4)	92 (53.5)		89 (52.0)	92 (53.8)	
secondary	3656 (43.6)	80 (46.5)		82 (48.0)	79 (46.2)	
**length of infertility, median (IQR)**	3.0 (2.0~4.5)	3.0 (2.0~5.0)	0.014	3.0 (2.0~5.0)	3.0 (2.0~5.0)	0.853
**main etiology, n (%)**			0.227			0.686
female factor	6374 (76.0)	130 (75.6)		133 (77.8)	129 (75.4)	
male factor	822 (9.8)	22 (12.8)		23 (13.5)	22 (12.9)	
male and female factors	894 (10.7)	18 (10.5)		12 (7.0)	18 (10.5)	
unexplained	301 (3.6)	2 (1.2)		3 (1.8)	2 (1.2)	
**number of oocytes retrieved, median (IQR)**	12 (7~17)	17 (12~20)	<0.001	17 (12~22)	17 (12~20)	0.830
**number of previous thawing, n (%)**			0.602			0.579
0	7051 (84.0)	142 (82.6)		137 (80.1)	141 (82.5)	
≥1	1340 (16.0)	30 (17.4)		34 (19.9)	30 (17.5)	
**development stage of embryo transferred, n (%)**			0.001			0.445
cleavage embryo	1710 (20.4)	17 (9.9)		13 (7.6)	17 (9.9)	
blastocyst	6681 (79.6)	155 (90.1)		158 (92.4)	154 (90.1)	
**endometrial preparation method, n (%)**			0.748			0.817
artificial	7027 (83.7)	147 (85.5)		150 (87.7)	146 (85.4)	
natural	445 (5.3)	7 (4.1)		6 (3.5)	7 (4.1)	
others	919 (11.0)	18 (10.5)		15 (8.8)	18 (10.5)	
**thickness of endometrium, median (IQR)**	9.1 (8.4~10.0)	9.0 (8.4~10.0)	0.737	9.1 (8.7~10.2)	9.0 (8.4~10.0)	0.174

OPU, ovum pick-up; IQR, interquartile range.

#### Primary Outcomes Before and After Matching

We mainly compared the CPR and LBR between two groups (storage time ≤1 year and 1-3 years, 3-5 years respectively) before and after matching. Results are shown in **Supplementary STable4 and STable5**. The CPR and LBR in patient with storage time ≤1 year and 1-3 years were comparable before and after matching. The patients with storage time 3-5 years had a higher CPR (54.0% vs. 47.5%, p=0.011) and LBR (42.2% vs. 37.1%, p=0.04) than patients with storage time ≤1 year before matching. After matching, they were statistically similar across two groups.

The comparisons between the Group ≤1 year and the Group >5 years are shown in **Table 6**. Before matching, no differences were found in CPR and LBR. However, the CPR (49.1% vs. 61.4%, p=0.022) and LBR (37.4% vs. 52.0%, p=0.007) were significantly lower in patients from Group >5 years after matching.

**Table 6:** The results and odds ratio of CPR and LBR in Group ≤1 year and Group >5 years before and after matching.

storage time	unmatched			matched		
	≤1 y	>5 y	p value	≤1 y	>5 y	p value
**CPR, n (%)**	3982 (47.5)	84 (48.8)	0.719	105 (61.4)	84 (49.1)	0.022
**LBR, n (%)**	3114 (37.1)	64 (37.2)	0.979	89 (52.0)	64 (37.4)	0.007
**CPR**						
crude	ref	1.057 (0.781,1.430)	0.719	ref	0.610 (0.388,0.960)	0.033
adjusted	ref	0.698 (0.511,0.954)	0.024	ref	0.566 (0.358,0.897)	0.015
**LBR**						
crude	ref	1.004 (0.735,1.372)	0.979	ref	0.548 (0.349,0.860)	0.009
adjusted	ref	0.646 (0.468,0.891)	0.008	ref	0.505 (0.319,0.799)	0.004

CPR, clinical pregnancy rate; LBR, live birth rate.

#### The Relationship Between Storage Time and Primary Outcomes Before and After Matching

The results of multivariable logistic regression of primary outcomes between the Group ≤1 year and Group 1-3, Group 3-5 years are presented in **Supplementary STable4** and **STable5**. After adjustment for the factors mentioned in baseline characteristics, the association between CPR or LBR and storage time did not reach statistical differences before and after matching.

**Table 6** indicates that CPR and LBR decreased with increasing of the storage time from ≤1 year to >5 years after eliminating the influence of baseline characteristics regardless of matching.

## Discussion

### Principal Findings

In this study, we included 9806 frozen-thawed cycles following single embryo transfer to analyze the correlation between storage time and pregnancy outcomes, and then screened out 3602 cycles to compare the perinatal outcomes among groups. Finally, we found that there were no significant differences in pregnancy outcomes when we stratified all cycles into 3 groups. Subgroup analysis based on maternal age at OPU also showed no significant differences. Moreover, postponement of transfer increased the risks of LGA and placenta previa after adjusting for confounding factors. In addition, comparisons of primary outcomes between the Group ≤1 year and Group 1-3, Group 3-5 years were analyzed showing no significant differences. However, deferral of embryo transfer beyond 5 years decreased CPR and LBR.

### Comparison With Other Studies

In theory, embryos can be preserved indefinitely in liquid nitrogen (-196°C) on account of inhibited enzyme activity and stagnant metabolism (1). The study of Riggs et al. (13) found that there was no significant influence of cryopreservation storage duration on post-thawing survival rate and pregnancy outcomes after analyzing 11768 FET cycles in IVF or oocyte donation patients between 1986 and 2007. Liu et al. (15) reported that the survival and pregnancy outcomes of slow-frozen cleavage-stage embryos were not influenced by storage time ranged from 12 to 119 months. However, some previous studies reached the opposite conclusions. Testart et al. (23) found that there was a progressive decrease in percentage of surviving embryonic cells with increase of time between freezing and thawing. All embryos utilized in the studies aforementioned were sampled using slow freezing methods, which was not a currently mainstream technique. In terms of vitrification, a retrospective study of Wirleitner et al. (16) showed that storage time had no negative impact on blastocyst survival and the implantation potential of embryos up to 6 years in all 603 cycles. But limitations such as including cycles only of blastocysts transfer and small sample size must be considered. A more recent study of 8736 single vitrified–warmed blastocyst transfer cycles was conducted by Ueno et al. (24) to investigate the impact of the duration of vitrification on pregnancy and neonatal outcomes. No significant differences among groups were detected. However, he only focused on the patients aged 35-39 years when picking oocytes and blastocyst transfer cycles, which may cause selection bias. There were also several studies (14, 25, 26) comparing the reproductive outcomes between the immediate and delayed thawing cycles for vitrified embryos following a freeze-all strategy or a failed fresh embryo transfer. The pregnancy outcomes of the immediate cycles were comparable to the delayed cycles. Most studies showed that vitrification did not affect pregnancy outcomes, but the results were still divided. A recent retrospective study performed by Li et al. (21) revealed that the time of cryopreservation negatively affected pregnancy outcomes, but did not influence neonatal outcomes among 24698 patients with the first vitrified embryo transfer following a freeze-all strategy. Therefore, more studies need to be conducted. Our study, which included 9806 frozen-thawed cycles, was conducted to evaluate the effect of vitrification time on both cleavage-stage embryos and blastocysts to provide clinical evidence.

The oocyte quality is one of the determinants of female fertility. The age-associated oxidative damage can lead to accumulation of mitochondrial deficits (27, 28). Damage to the spindle may lead an increase in formation of aneuploid gametes (29). A recent study of Sun et al. (27) showed that CPR and LBR decreased while the miscarriage rate increased significantly with increase of age for embryo transfer, especially after the age of 34 years. In 4958 infertile women using a freeze-all strategy, Lin et al. (30) concluded that LBR for the first FET cycle significantly decreased with increasing maternal age. The study conducted by Zhang et al. (31) found that increasing maternal age had an adverse impact on LBR in 256,643 fresh ART cycles. Therefore, age can be considered as an independent risk factor significantly affecting the pregnancy outcomes. To further elaborate on the relationship between the storage time and primary outcome measures, subgroup analysis was performed according to the maternal age at OPU (<30, 30-35, ≥35 years old). Our study showed that the storage time had no significant effect on CPR and LBR after adjustment in all subgroups. The findings of our study could enrich the evidence in this field and may help the clinicians in clinical decision making.

A multicenter randomized controlled trial conducted by Wei et al. (32) indicated that frozen single blastocyst transfer was associated with higher risks of pre-eclampsia and LGA compared to fresh cycles. The CoNARTaS group performed a study to investigate the differences in birth weight between singletons born after FET (n=17500) and fresh ET (n=69510). The results showed that all singletons born after FET had a higher risk of LGA. After analyzing 277,042 single-embryo transfer cycles, Ishihara et al. (33) presented that FET was associated with a higher incidence of LGA, placenta complication and pregnancy-induced hypertension. Our results are in general agreement with previously published studies. The risks of LGA and placenta previa may be related to the process of FET and even to the storage time of vitrification. The underlying mechanisms of these changes were still largely unclear. However, several studies (34–36) supported that the alternation of epigenetics in the process of FET was linked with abnormal placentation and fetal growth. In addition, differences in gene expression were detected in placentas and fetuses from FET cycles (37). Owing to the limited sample size and multiple potential factors during pregnancy, further researches are needed to address these changes.

Many cases of successful pregnancy and delivery of healthy fetuses were reported globally after cryopreserving for a long period of time. Revel et al. (38) reported the delivery of healthy twins following the transfer of four embryos in storage for 12 years. A healthy live birth, which was recorded by Dowling-Lacey et al. (39), was achieved after transfer of pronuclear stage frozen embryos that were cryopreserved for almost 20 years. In addition, in 2017, the embryo which underwent the longest frozen duration around 25 years gave live birth successfully in the United States. However, these case reports could not represent the total patients in FET treatments. The study from Liu et al. (15) presented that cryopreservation for more than 48 months did not affect the survival and pregnancy outcomes. Another retrospective study conducted by Wirleitner et al. (16) showed no negative effects of prolonged storage for up to 6 years. However, the limitation of sample size posed concern about reliability of the results. Therefore, we were proposing whether freezing for more than 3 years or even more than 5 years would affect the primary outcomes. A striking feature in our study was 1:1 propensity score matching, carried out to eliminate characteristic differences between groups when a large gap in the sample size was present. We filtered out embryos stored for 1-3 years, 3-5 years and >5 years to compare with embryos stored for ≤1 year respectively. A multivariate logistic regression model was further established to explore the association between the storage time and primary outcomes. Finally, we found that the CPR and LBR decreased significantly when the duration of vitrification exceeded 5 years. Potential explanations were proposed as followed. First, high-concentration cryoprotectants may induce cellular damage, which includes both direct biochemical toxicity and osmotic injury (10, 12). Second, the Cryotop we use forms an “open system”, which makes the embryos contact with liquid nitrogen directly during storage (12). Third, accidental warming caused by the movement of samples in and out of liquid nitrogen tanks may influence embryos in the same cannister, even if they are not removed themselves (10).

### Strengths and Limitations

To our knowledge, this study possessed the largest sample size to date to explore the time limit of vitrification. Additionally, this is the first study to evaluate the effect of storage time to the primary outcomes in different age groups individually. This study also had several limitations. First, it was a retrospective study conducted in a single medicine center. Selection bias and confounders might exist inevitably. Second, we did not proceed a long-term follow-up to provide more convincing evidence about offspring. In addition, it is noteworthy to point out that a group of 171 cycles remained a small sample size after matching, expansion of sample size is needed for future data verification.

### Implications

Over recent years, with improvement of ovarian stimulation protocol as well as emphasis on fertility preservation, there has been a surge in FET treatments. Still, safety remains a focus of concern. Therefore, it is very important to explore the impact of storage time on pregnancy outcomes and perinatal outcomes. With sufficient evidence, clinicians could improve embryo utilization and achieve a better pregnancy outcome. Such results may affect the clinical decision making by experts, such as the time of embryo transfer and the time limit of cryopreservation.

## Conclusion

In summary, within 5 years of storage time, pregnancy outcomes were not significantly affected. Subgroup analysis according to the maternal age at OPU further confirmed the results. However, CPR and LBR decreased significantly when the storage time exceeded 5 years. It is worth noting that this result was drawn from a small sample size from different time periods, so it should be interpreted with caution and confirmed with larger sample sizes in future studies. In addition, deferral of embryo transfer increased the risks of LGA and placental complications. As a steep rise in FET treatments, the findings of our study enriched the evidence of the association between storage time and pregnancy outcomes, subsequently provided a novel insight into the discussion about time limit of vitrification.

## Data Availability Statement

The original contributions presented in the study are included in the article/**Supplementary Material**. Further inquiries can be directed to the corresponding authors.

## Ethics Statement

This study involving human participants was reviewed and approved by Institution Review Board (IRB) of Tongji Hospital. The patients/participants provided their written informed consent to participate in this study.

## Author Contributions

MC contributed to the data analysis and manuscript preparation. XD performed the data collection. SL participated in the data analysis and manuscript revision. YZ and JA were involved in study design and patient recruitment. All authors read and approved the final manuscript.

## Conflict of Interest

The authors declare that the research was conducted in the absence of any commercial or financial relationships that could be construed as a potential conflict of interest.

## Publisher’s Note

All claims expressed in this article are solely those of the authors and do not necessarily represent those of their affiliated organizations, or those of the publisher, the editors and the reviewers. Any product that may be evaluated in this article, or claim that may be made by its manufacturer, is not guaranteed or endorsed by the publisher.
