# Supplementary material for: The Impact of Embryo Storage Time on Pregnancy and Perinatal Outcomes and the Time Limit of Vitrification: A Retrospective Cohort Study
Source: Front Endocrinol (Lausanne). 2021 Oct 25;12:724853. doi: 10.3389/fendo.2021.724853 (PMC8579853; doi:10.3389/fendo.2021.724853)
Supplement: Supplementary file 1 [file DataSheet_1.docx]

STable1. Subgroup analysis of CPR and LBR according to maternal age at OPU.

|  | **N** |  |  | **p value** | **0-3 m** | | **3-12 m** | | **>12 m** |
| --- | --- | --- | --- | --- | --- | --- | --- | --- | --- |
|  |  |  |  |  | **OR (95% CI)** | **p value** | **OR (95% CI)** | **p value** | **ref** |
| **maternal age at OPU** | | |  |  |  |  |  |  |  |
| **<30** | 3513 | CPR | crude | 0.946 | 0.972(0.814,1.162) | 0.758 | 0.990(0.818,1.198) | 0.915 | 1 |
|  |  |  | adjusted | 0.132 | 0.911(0.755,1.100) | 0.335 | 1.080(0.888,1.314) | 0.442 | 1 |
|  |  | LBR | crude | 0.772 | 1.067(0.894,1.273) | 0.473 | 1.044(0.864,1.261) | 0.659 | 1 |
|  |  |  | adjusted | 0.336 | 1.012(0.841,1.219) | 0.899 | 1.128(0.930,1.369) | 0.221 | 1 |
| **≥30, <35** | 3354 | CPR | crude | 0.915 | 1.013(0.820,1.252) | 0.902 | 0.982(0.789,1.222) | 0.871 | 1 |
|  |  |  | adjusted | 0.849 | 1.002(0.801,1.254) | 0.985 | 1.046(0.837,1.308) | 0.693 | 1 |
|  |  | LBR | crude | 0.246 | 1.164(0.938,1.445) | 0.169 | 1.053(0.842,1.318) | 0.648 | 1 |
|  |  |  | adjusted | 0.505 | 1.146(0.911,1.440) | 0.244 | 1.114(0.887,1.399) | 0.354 | 1 |
| **≥35** | 2939 | CPR | crude | 0.926 | 1.046(0.797,1.371) | 0.747 | 1.020(0.772,1.348) | 0.889 | 1 |
|  |  |  | adjusted | 0.102 | 1.296(0.968,1.734) | 0.082 | 1.116(0.828,1.504) | 0.470 | 1 |
|  |  | LBR | crude | 0.892 | 1.077(0.787,1.472) | 0.644 | 1.077(0.781,1.485) | 0.652 | 1 |
|  |  |  | adjusted | 0.164 | 1.350(0.969,1.882) | 0.076 | 1.206(0.859,1.694) | 0.279 | 1 |

OPU: ovum pick-up, CPR: clinical pregnancy rate, LBR: live birth rate, OR: odds ratio, CI: confidence interval.

STable2. Baseline characteristics of patients with storage time ≤1 year and >1, ≤3 years before and after matching.

|  | **unmatched** | | | **matched** | | |
| --- | --- | --- | --- | --- | --- | --- |
| **storage time** | **≤1 y** | **>1 y, ≤3 y** | **p value** | **≤1 y** | **>1 y, ≤3 y** | **p value** |
| **number of cycles, n** | 8391 | 845 |  | 844 | 844 |  |
| **maternal age at OPU, median (IQR)** | 32(28~36) | 31(28~35) | 0.001 | 32(28~35) | 31(28~35) | 0.127 |
| **maternal BMI, median (IQR)** | 21.5(19.8~23.6) | 21.9(19.9~24.0) | 0.003 | 21.8(20.0~24.3) | 21.9(19.9~24.0) | 0.730 |
| **type of infertility, n (%)** |  |  | 0.169 |  |  | 1.000 |
| primary | 4735(56.4) | 456(54.0) |  | 455(53.9) | 455(53.9) |  |
| secondary | 3656(43.6) | 389(46.0) |  | 389(46.1) | 389(46.1) |  |
| **length of infertility, median (IQR)** | 3.0(2.0~4.5) | 3.0(2.0~5.0) | 0.017 | 3.0(2.0~5.0) | 3.0(2.0~5.0) | 0.799 |
| **main etiology, n (%)** |  |  | 0.112 |  |  | 0.308 |
| female factor | 6374(76.0) | 668(79.1) |  | 691(81.9) | 667(79.0) |  |
| male factor | 822(9.8) | 81(9.6) |  | 60(7.1) | 81(9.6) |  |
| male and female factors | 894(10.7) | 75(8.9) |  | 73(8.6) | 75(8.9) |  |
| unexplained | 301(3.6) | 21(2.5) |  | 20(2.4) | 21(2.5) |  |
| **number of oocytes retrieved,**  **median (IQR)** | 12(7~17) | 13(8~18) | <0.001 | 12(8~18) | 13(8~18) | 0.127 |
| **number of previous thawing, n (%)** |  |  | <0.001 |  |  | 1.000 |
| 0 | 7051(84.0) | 484(57.3) |  | 484(57.3) | 484(57.3) |  |
| ≥1 | 1340(16.0) | 361(42.7) |  | 360(42.7) | 360(42.7) |  |
| **development stage of embryo transferred, n (%)** |  |  | 0.001 |  |  | 0.691 |
| cleavage embryo | 1710(20.4) | 133(15.7) |  | 139(16.5) | 133(15.8) |  |
| blastocyst | 6681(79.6) | 712(84.3) |  | 705(83.5) | 711(84.2) |  |
| **endometrial preparation method,**  **n (%)** |  |  | 0.001 |  |  | 0.640 |
| artificial | 7027(83.7) | 666(78.8) |  | 657(77.8) | 666(78.9) |  |
| natural | 445(5.3) | 64(7.6) |  | 60(7.1) | 64(7.6) |  |
| others | 919(11.0) | 115(13.6) |  | 127(15.0) | 114(13.5) |  |
| **thickness of endometrium,**  **median (IQR)** | 9.1(8.4~10.0) | 9.0(8.2~10.0) | 0.004 | 9.0(8.3~9.9) | 9.0(8.2~10.0) | 0.730 |

OPU: ovum pick-up, IQR: interquartile range.

STable3. Baseline characteristics of patients with storage time ≤1 year and >3, ≤5 years before and after matching.

|  | **unmatched** | | | **matched** | | |
| --- | --- | --- | --- | --- | --- | --- |
| **storage time** | **≤1 y** | **>3 y, ≤5 y** | **p value** | **≤1 y** | **>3 y, ≤5 y** | **p value** |
| **number of cycles, n** | 8391 | 398 |  | 398 | 398 |  |
| **maternal age at OPU, median (IQR)** | 32(28~36) | 29(26~31) | <0.001 | 29.0(26.8~31.0) | 29.0(26.0~31.0) | 0.488 |
| **maternal BMI, median (IQR)** | 21.5(19.8~23.6) | 21.3(19.6~23.5) | 0.381 | 21.5(19.5~23.5) | 21.3(19.6~23.5) | 0.987 |
| **type of infertility, n (%)** |  |  | 0.034 |  |  | 0.468 |
| primary | 4735(56.4) | 246(61.8) |  | 236(59.3) | 246(61.8) |  |
| secondary | 3656(43.6) | 152(38.2) |  | 162(40.7) | 152(38.2) |  |
| **length of infertility, median (IQR)** | 3.0(2.0~4.5) | 3.0(2.0~5.0) | 0.086 | 3.0(2.0~5.0) | 3.0(2.0~5.0) | 0.695 |
| **main etiology, n (%)** |  |  | 0.404 |  |  | 0.726 |
| female factor | 6374(76.0) | 291(73.1) |  | 278(69.8) | 291(73.1) |  |
| male factor | 822(9.8) | 46(11.6) |  | 52(13.1) | 46(11.6) |  |
| male and female factors | 894(10.7) | 49(12.3) |  | 57(14.3) | 49(12.3) |  |
| unexplained | 301(3.6) | 12(3.0) |  | 11(2.8) | 12(3.0) |  |
| **number of oocytes retrieved,**  **median (IQR)** | 12.0(7.0~17.0) | 15.0(12.0~20.3) | <0.001 | 16.0(11.0~22.0) | 15.0(12.0~20.3) | 0.726 |
| **number of previous thawing, n (%)** |  |  | 0.127 |  |  | 0.714 |
| 0 | 7051(84.0) | 323(81.2) |  | 327(82.2) | 323(81.2) |  |
| ≥1 | 1340(16.0) | 75(18.8) |  | 71(17.8) | 75(18.8) |  |
| **development stage of embryo transferred, n (%)** |  |  | <0.001 |  |  | 0.597 |
| cleavage embryo | 1710(20.4) | 33(8.3) |  | 29(7.3) | 33(8.3) |  |
| blastocyst | 6681(79.6) | 365(91.7) |  | 369(92.7) | 365(91.7) |  |
| **endometrial preparation method,**  **n (%)** |  |  | <0.001 |  |  | 0.054 |
| artificial | 7027(83.7) | 353(88.7) |  | 352(88.4) | 353(88.7) |  |
| natural | 445(5.3) | 29(7.3) |  | 18(4.5) | 29(7.3) |  |
| others | 919(11.0) | 16(4.0) |  | 28(7.0) | 16(4.0) |  |
| **thickness of endometrium,**  **median (IQR)** | 9.1(8.4~10.0) | 9.1(8.4~10.1) | 0.740 | 9.2(8.5~10.0) | 9.1(8.4~10.1) | 0.423 |

OPU: ovum pick-up, IQR: interquartile range.

STable4. The results and odds ratio of CPR and LBR in Group ≤1 year and Group >1, ≤3 years.

|  | **unmatched** | | | **matched** | | |
| --- | --- | --- | --- | --- | --- | --- |
| **storage time** | **≤1 y** | **>1 y, ≤3 y** | **p value** | **≤1 y** | **>1 y, ≤3 y** | **p value** |
| **CPR, n (%)** | 3982(47.5) | 416(49.2) | 0.325 | 389(46.1) | 416(49.3) | 0.188 |
| **LBR, n (%)** | 3114(37.1) | 313(37.0) | 0.968 | 295(35.0) | 313(37.1) | 0.361 |
| **CPR** |  |  |  |  |  |  |
| crude | ref | 1.074(0.932,1.237) | 0.325 | ref | 1.137(0.939,1.376) | 0.188 |
| adjusted | ref | 1.044(0.897,1.217) | 0.577 | ref | 1.116(0.913,1.365) | 0.285 |
| **LBR** |  |  |  |  |  |  |
| crude | ref | 0.997(0.861,1.154) | 0.968 | ref | 1.097(0.899,1.338) | 0.361 |
| adjusted | ref | 0.962(0.822,1.127) | 0.633 | ref | 1.060(0.861,1.305) | 0.585 |

CPR: clinical pregnancy rate, LBR: live birth rate.

STable5. The results and odds ratio of CPR and LBR in Group ≤1 year and Group >3, ≤5 years.

|  | **unmatched** | | | **matched** | | |
| --- | --- | --- | --- | --- | --- | --- |
| **storage time** | **≤1 y** | **>3 y, ≤5 y** | **p value** | **≤1 y** | **>3 y, ≤5 y** | **p value** |
| **CPR, n (%)** | 3982(47.5) | 215(54.0) | 0.010 | 213(53.5) | 215(54.0) | 0.887 |
| **LBR, n (%)** | 3114(37.1) | 168(42.2) | 0.040 | 167(42.0) | 168(42.2) | 0.943 |
| **CPR** |  |  |  |  |  |  |
| crude | ref | 1.301(1.063,1.592) | 0.011 | ref | 1.020(0.772,1.348) | 0.887 |
| adjusted | ref | 0.891(0.722,1.099) | 0.281 | ref | 1.051(0.787,1.403) | 0.736 |
| **LBR** |  |  |  |  |  |  |
| crude | ref | 1.238(1.010,1.518) | 0.040 | ref | 1.010(0.763,1.339) | 0.943 |
| adjusted | ref | 0.831(0.672,1.027) | 0.087 | ref | 1.022(0.764,1.367) | 0.883 |

CPR: clinical pregnancy rate, LBR: live birth rate.
